# Elevated Circulating Ceramides 18:0 and 24:1 as a Risk Factor for Sarcopenia: In Vitro, Animal, and Clinical Evidence

**DOI:** 10.1002/jcsm.70310

**Published:** 2026-05-01

**Authors:** So Jeong Park, Ji Yeon Baek, Shibo Wei, Jin Young Lee, Yuna Lee, Sang‐Hoon Lee, Il‐Young Jang, Hyuk Sakong, Hyun Ju Yoo, Hee‐Won Jung, Eunju Lee, Su Jung Kim, Yunju Jo, Kyunggon Kim, Dongryeol Ryu, Beom‐Jun Kim

**Affiliations:** ^1^ Asan Institute for Life Sciences Asan Medical Center, University of Ulsan College of Medicine Seoul South Korea; ^2^ Division of Geriatrics, Department of Internal Medicine Asan Medical Center, University of Ulsan College of Medicine Seoul South Korea; ^3^ Department of Biomedical Science and Engineering Gwangju Institute of Science and Technology Gwangju South Korea; ^4^ Department of Convergence Medicine, Asan Institute for Life Sciences Asan Medical Center, University of Ulsan College of Medicine Seoul South Korea; ^5^ Department of Microbiology Wonkwang University School of Medicine Iksan South Korea; ^6^ Department of Digital Medicine, BK21 Project University of Ulsan College of Medicine Seoul South Korea; ^7^ Division of Endocrinology and Metabolism, Department of Internal Medicine Asan Medical Center, University of Ulsan College of Medicine Seoul South Korea

**Keywords:** biomarker, ceramides, oxidative stress, sarcopenia, therapeutic target

## Abstract

**Background:**

Ceramides have garnered considerable attention as pro‐aging bioactive lipids implicated in both metabolic dysfunction and musculoskeletal decline. Among these, C18:0 and C24:1 ceramides may play a role in the pathophysiology of sarcopenia, a key manifestation of age‐related deterioration. However, their specific contributions to muscle degeneration remain poorly defined.

**Methods:**

C2C12 myoblasts and primary myoblasts were treated with C18:0 or C24:1 ceramides during differentiation to assess myotube formation, migration and intracellular reactive oxygen species (ROS) levels. Three‐month‐old C57BL/6 mice received daily intraperitoneal injections of C18:0 or C24:1 ceramides for 4 weeks to evaluate muscle morphology and function. In a human cohort of 165 community‐dwelling older adults (≥ 65 years), serum ceramide levels were measured via LC–MS/MS and analysed in relation to sarcopenia parameters.

**Results:**

Both C18:0 and C24:1 ceramides significantly impaired myogenic differentiation in vitro, as evidenced by reduced myotube number, total myotube area, average area per myotube, nuclei count per myotube and fusion index, through ROS‐mediated mechanisms (with up to an 8.6‐fold increase in ROS production). Consistently, C18:0 and C24:1 ceramides markedly downregulated key myogenic markers and inhibited ITGB1‐FAK‐AKT signalling while promoting nuclear activation of FoxO‐associated catabolic pathways. These deleterious effects were attenuated by treatment with the antioxidant N‐acetylcysteine. In mice, systemic administration of either ceramide resulted in reduced muscle fibre cross‐sectional area in the tibialis anterior (by 20.5% and 20.9% for C18:0 and C24:1, respectively) and soleus muscles (by 18.1% and 16.1%), accompanied by decreased grip strength, shorter grid hanging times and reduced latency to fall in the rotarod test. Clinically, in a cohort of 165 older adults (80.6% female; mean age 75.2 ± 5.2 years in controls and 79.7 ± 4.8 years in the sarcopenia group), serum levels of C18:0 and C24:1 ceramides were 27% and 14% higher, respectively, in individuals with sarcopenia compared to controls (*p* = 0.001 and 0.018). Furthermore, each standard deviation increase in serum C18:0 and C24:1 ceramide levels was associated with a 2.0‐ and 1.6‐fold increased risk of sarcopenia, respectively (*p* = 0.003 and 0.040).

**Conclusions:**

Our findings reveal that circulating C18:0 and C24:1 ceramides are significantly associated with sarcopenia in older adults, while experimental models demonstrate they promote muscle atrophy through oxidative stress‐induced impairment of myogenesis and muscle function. These ceramides may serve as minimally invasive biomarkers and potential therapeutic targets for age‐related muscle decline. Interventions aimed at modulating ceramide metabolism could offer new avenues for sarcopenia prevention and treatment in aging populations.

## 1 Introduction

Aging is characterised by progressive physiological decline across multiple tissues and is driven by both intrinsic and extrinsic mechanisms, including systemic factors present in the circulation [1]. Heterochronic parabiosis models, which surgically conjoin the circulatory systems of young and old animals, have provided compelling evidence that blood‐borne factors play a pivotal role in regulating tissue aging [2]. Exposure of young animals to an aged circulatory environment accelerates functional deterioration, whereas aged animals benefit from exposure to youthful blood, suggesting the presence of pro‐aging and pro‐youthful circulating factors [3, 4]. These findings have catalysed research aimed at identifying specific molecules within the systemic milieu that mediate such age‐related changes, with an increasing focus on their implications for age‐associated diseases [5].

Among the most affected systems during aging are bone and skeletal muscle, which engage in profound mechanical and biochemical crosstalk [6, 7]. The concurrent deterioration of these tissues—manifesting as osteoporosis and sarcopenia—is highly prevalent in older adults, significantly elevating the risk of falls and disability [6, 8]. Given their shared risk factors, identifying common biological pathways that drive both conditions is crucial for developing effective preventive and therapeutic strategies.

Ceramides, a class of sphingolipids, are increasingly recognised as bioactive lipotoxic mediators involved in numerous age‐related disorders, including cardiovascular disease, insulin resistance and cognitive decline [9, 10, 11]. Recent evidence has demonstrated that certain ceramide species, such as C18:0 and C24:1 ceramides, increase with age and exert detrimental effects on bone metabolism by inducing senescence in human bone marrow stromal cells, promoting osteoclastogenesis, and enhancing bone resorption, thereby contributing to osteoporosis and fragility fractures in the elderly [12, 13]. However, despite the well‐established pathophysiological link between osteoporosis and sarcopenia [6, 14], it remains unknown whether these ‘osteotoxic’ ceramides exert similar deleterious effects on skeletal muscle. To address this gap, we employed a translational approach combining in vitro and animal models to elucidate the direct impact of C18:0 and C24:1 ceramides on muscle metabolism, while also validating their clinical significance as potential biomarkers for sarcopenia in an older adult cohort.

## 2 Materials and Methods

### 2.1 Cell Culture and Reagents

Mouse C2C12 myoblasts (American Type Culture Collection, Manassas, Virginia, United States) were propagated in Dulbecco's modified Eagle's medium (DMEM) supplemented with 15% foetal bovine serum (FBS), 20 mM HEPES, 2 mM L‐glutamine, 100 U/mL penicillin and 0.1 mg/mL streptomycin (all from Life Technologies, Carlsbad, California, United States). Cells were maintained in a humidified incubator at 37 °C with 5% CO_2_. For induction of myogenic differentiation, cells were allowed to reach approximately 90% confluency in the growth medium, followed by replacement with differentiation medium composed of DMEM containing 2% horse serum. Differentiation was carried out over 3 to 4 days. Synthetic C18:0 and C24:1 ceramides were purchased from Cayman Chemical (Ann Arbor, Michigan, United States).

### 2.2 Animals

All animal procedures were approved by the Institutional Animal Care and Use Committee of the Asan Institute for Life Sciences (Protocol No. 2019‐12‐143). Male C57BL/6 mice, aged 3 months, were purchased from Orientbio (Seongnam, South Korea) and used for all experiments. Mice were randomly assigned to receive intraperitoneal injections of C18:0 ceramide (50 μg/100 μL), C24:1 ceramide (50 μg/100 μL) or PBS (100 μL) using a 31‐gauge needle, administered five times per week for a total of 4 weeks (*n* = 6 per group). All groups were weight‐matched at baseline. Mice were euthanised at 16 weeks of age by cardiac puncture under anaesthesia. While the researcher administering the treatments was aware of group assignments, outcome assessors measuring muscle parameters were blinded to the intervention groups.

### 2.3 Study Participants for Clinical Research

This clinical study recruited Korean adults aged ≥ 65 years who underwent comprehensive geriatric assessments at the Division of Geriatrics or Endocrinology, Department of Internal Medicine, Asan Medical Center (AMC), Seoul, Korea, between May 2020 and March 2021. All participants were ambulatory, community‐dwelling individuals who visited the outpatient clinic for non‐specific complaints such as fatigue or appetite loss, or for the management of chronic conditions including osteoarthritis, hypertension and hyperlipidaemia. Individuals residing in long‐term care facilities or admitted to the hospital were excluded. Additional exclusion criteria included end‐stage renal disease, active malignancy and symptomatic heart failure with a life expectancy of less than 1 year. A total of 165 eligible individuals provided written informed consent and underwent blood sampling for sarcopenia‐related assessments during their outpatient visit. The study protocol was approved by the Institutional Review Board of AMC (IRB No. 2020‐0524) and conducted in accordance with the principles of the Declaration of Helsinki. This study was reported in accordance with the STROBE guidelines.

### 2.4 Additional Information for In Vitro, Animal and Clinical Research

Detailed information is provided in the Supplementary Materials and Methods.

### 2.5 Statistical Analysis

For experimental data, results are expressed as mean ± standard error of the mean (SEM) from at least three independent experiments, each performed in triplicate, unless otherwise specified. Comparisons among three or more groups were assessed using one‐way analysis of variance (ANOVA) followed by Tukey's post hoc test. For comparisons between two groups, the Mann–Whitney *U* test was applied.

Clinical data are presented as mean ± standard deviation (SD), or as frequencies and percentages where appropriate. Baseline characteristics between participants with and without sarcopenia were compared using the Student's *t* test for continuous variables and the chi‐square test for categorical variables. Analysis of covariance (ANCOVA) was used to compare estimated mean serum ceramide levels according to sarcopenia status and related parameters, with adjustment for age, sex, and body mass index (BMI). Logistic regression analysis was conducted to calculate odds ratios (ORs) and 95% confidence intervals (CIs) for the association between serum ceramide levels and the risk of sarcopenia or adverse muscle outcomes. All statistical analyses were performed using SPSS version 18.0 (SPSS Inc., Chicago, Illinois, United States). A two‐tailed *p* value of < 0.05 was considered statistically significant.

## 3 Results

### 3.1 Inhibitory Effects of C18:0 and C24:1 Ceramides on In Vitro Myogenesis via Increased Intracellular ROS

**FIGURE 1:**
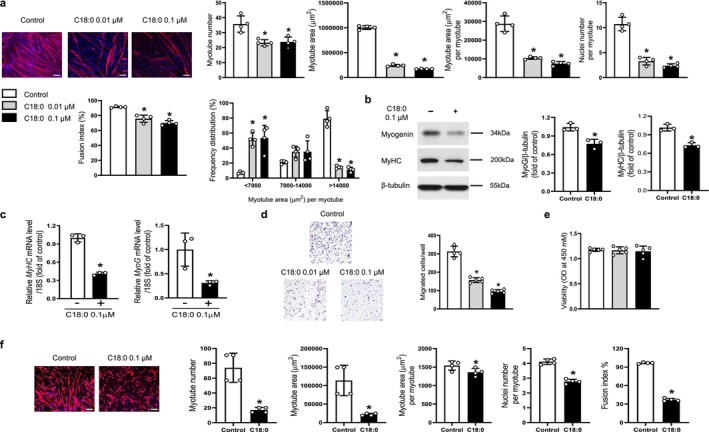
C18:0 ceramide impairs in vitro myogenic differentiation. (a) Mouse C2C12 myoblasts were differentiated into myotubes using 2% horse serum in the presence of the indicated concentrations of C18:0 ceramide for 3 days. Myotubes were immunostained with an anti‐MyHC antibody, and nuclei were counterstained with DAPI. Quantitative analyses per field are shown (*n* = 4). (b) Western blot and (c) quantitative reverse transcription polymerase chain reaction analyses were performed to assess the expression levels of myogenic markers myogenin and MyHC following 3‐day treatment with 0.1 μM C18:0 ceramide during differentiation (*n* = 3). (d) Myoblast migration was evaluated using a Boyden chamber assay, and (e) cell viability was measured with a CCK‐8 assay after treatment with the indicated concentrations of C18:0 ceramide for 6 and 24 h, respectively (*n* = 5). (f) The inhibitory effects of C18:0 ceramide on myogenic differentiation were similarly confirmed using primary myoblasts under the same experimental conditions as described in (a). Scale bars: 100 μm (a), 50 μm (d), 100 μm (f). MyHC, myosin heavy chain; DAPI, 4′,6‐diamidino‐2‐phenylindole; OD, optical density. *p < 0.05 vs. untreated control.

In vitro studies utilising the C2C12 myoblast cell line revealed that exposure to C18:0 ceramide at concentrations of 0.01 μM and 0.1 μM during the differentiation phase led to a consistent decrease in myotube number, total myotube area, average area per myotube, nuclei count per myotube and the fusion index (Figure 1a). Notably, the maximal concentration used in these assays (0.1 μM, equivalent to 100 nM) was carefully selected to closely reflect the pathologically relevant circulating levels observed in our human cohort (e.g., ~103.4 nM for C18:0 in sarcopenic patients) while avoiding non‐specific lipotoxicity in serum‐reduced culture conditions. This inhibitory effect was accompanied by a significant shift in the myotube size distribution, with a higher proportion of small‐diameter myotubes and a corresponding reduction in larger ones (Figure 1a). Consistent with these morphological findings, western blot and qRT‐PCR analyses demonstrated substantial downregulation of the key myogenic markers myogenin and MyHC following C18:0 ceramide treatment (Figure 1b,c). Additionally, C18:0 ceramide exerted a dose‐dependent suppressive effect on the migratory capacity of C2C12 myoblasts, as shown in the transwell assay, without adversely affecting cell viability (Figure 1d,e, respectively). Notably, a similar pattern of impaired myogenic differentiation was observed in primary myoblasts exposed to C18:0 ceramide, supporting the generalizability of these findings (Figure 1f).

**FIGURE 2:**
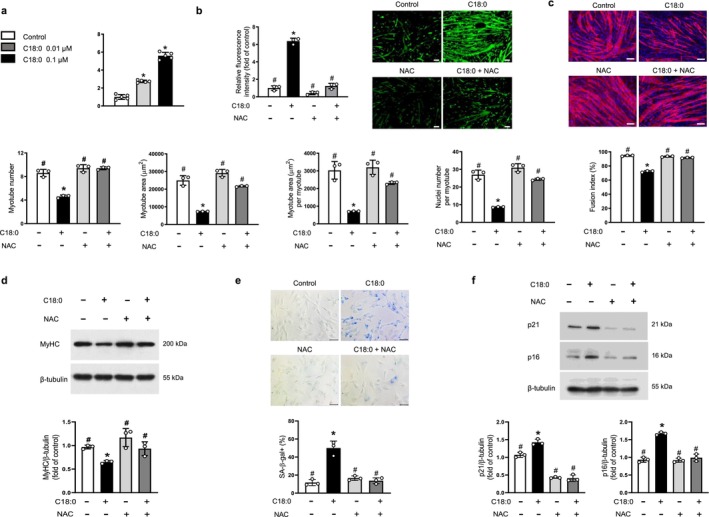
The inhibitory effects of C18:0 ceramide on myogenesis are mediated by increased intracellular ROS generation. (a) Mouse C2C12 myoblasts were differentiated into myotubes using 2% horse serum in the presence of the indicated concentrations of C18:0 ceramide for 3 days. Intracellular ROS levels were assessed using the fluorescent probe chloromethyl‐2′,7′‐dichlorofluorescein diacetate (CM‐H_2_DCFDA) (*n* = 5). (b,c) C2C12 cells were differentiated into myotubes with 2% horse serum in the presence or absence of 0.1 μM C18:0 ceramide and/or 1 mM NAC for 3 days. (b) Intracellular ROS levels were measured using H_2_DCFDA (*n* = 3). (c) Myotubes were stained with anti‐MyHC antibody, and nuclei were counterstained with DAPI. Quantitative analyses of myogenic parameters per field are shown (*n* = 3). (d) Expression level of MyHC was assessed by western blotting under the same conditions (*n* = 3). (e,f) C2C12 cells were treated with 0.1 μM C18:0 ceramide and/or 1 mM NAC for 1 day (*n* = 3). (e) SA‐β‐gal‐positive cells (%) per field were quantified, and representative images of SA‐β‐gal staining are shown. SA‐β‐gal‐positive cells appear blue. (f) Western blot analyses were performed to assess p21 and p16 expression levels. β‐Tubulin served as a loading control. Scale bars: 100 μm (b,c), 20 μm (e). ROS, reactive oxygen species; NAC, N‐acetyl cysteine; MyHC, myosin heavy chain; DAPI, 4′,6‐diamidino‐2‐phenylindole; SA‐β‐gal, senescence‐associated β‐galactosidase. *p < 0.05 vs. untreated control; #*p* < 0.05 vs. 0.1 μM C18:0 ceramide.

To explore the involvement of oxidative stress, intracellular ROS levels were assessed using CM‐H_2_DCFDA fluorescence. C18:0 ceramide exposure resulted in a marked, concentration‐dependent elevation in ROS production, with a 5.6‐fold increase in fluorescence intensity at 0.1 μM (Figure 2a). Pretreatment with N‐acetyl cysteine (NAC), a potent antioxidant, significantly mitigated this ROS accumulation (Figure 2b). Consistent with a causal role for ROS, NAC co‐treatment markedly rescued the C18:0 ceramide‐induced impairment in myotube formation, restoring myotube number, area metrics, and fusion index (Figure 2c), and reversed the suppression of MyHC expression (Figure 2d). Furthermore, we found that C18:0 ceramide induced cellular senescence of C2C12 myoblasts, as evidenced by an increase in SA‐β‐gal‐positive cells and elevated expression of the cell cycle arrest‐related proteins p21 and p16 (Figure 2e,f, respectively). Notably, these pro‐senescent effects of C18:0 ceramide were also significantly attenuated by NAC. Taken together, these results highlight oxidative stress as a key mediator of both the anti‐myogenic and pro‐senescent effects of C18:0 ceramide.

**FIGURE 3:**
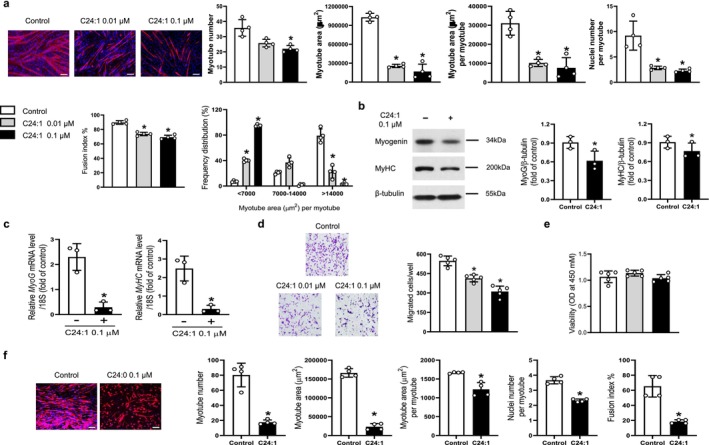
C24:1 ceramide impairs in vitro myogenic differentiation. (a) Mouse C2C12 myoblasts were differentiated into myotubes using 2% horse serum in the presence of the indicated concentrations of C24:1 ceramide for 3 days. Myotubes were immunostained with an anti‐MyHC antibody, and nuclei were counterstained with DAPI. Quantitative analyses per field are shown (*n* = 4). (b) Western blot and (c) quantitative reverse transcription polymerase chain reaction analyses were conducted to evaluate the expression of myogenic markers, myogenin and MyHC, following 3‐day treatment with 0.1 μM C24:1 ceramide during differentiation (*n* = 3). (d) Myoblast migration was assessed using a Boyden chamber assay, and (e) cell viability was measured using a CCK‐8 assay after treatment with the indicated concentrations of C24:1 ceramide for 6 and 24 h, respectively (*n* = 5). (f) The inhibitory effects of C24:1 ceramide on myogenic differentiation were also confirmed in primary myoblasts under the same experimental conditions as described in (a). Scale bars: 100 μm (a), 50 μm (d), 100 μm (f). MyHC, myosin heavy chain; DAPI, 4′,6‐diamidino‐2‐phenylindole; OD, optical density. *p < 0.05 vs. untreated control.

Comparable inhibitory effects were observed following treatment with C24:1 ceramide. Immunofluorescence staining, western blotting and qRT‐PCR confirmed that C24:1 ceramide significantly impaired myotube formation and downregulated myogenic markers in C2C12 cells (Figure 3a–c, respectively). Similar to C18:0, C24:1 ceramide also reduced C2C12 cell migration in a dose‐dependent manner without altering cell viability (Figure 3d,e, respectively). These anti‐myogenic effects were likewise validated in primary myoblasts (Figure 3f).

**FIGURE 4:**
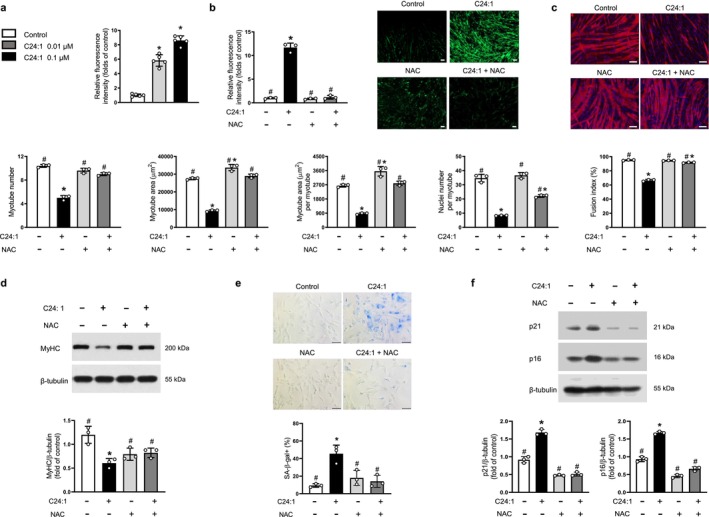
The inhibitory effects of C24:1 ceramide on myogenesis are mediated by increased intracellular ROS generation. (a) Mouse C2C12 myoblasts were differentiated into myotubes using 2% horse serum in the presence of the indicated concentrations of C24:1 ceramide for 3 days. Intracellular ROS levels were assessed using the fluorescent probe chloromethyl‐2′,7′‐dichlorofluorescein diacetate (CM‐H_2_DCFDA) (*n* = 5). (b,c) C2C12 cells were differentiated into myotubes with 2% horse serum in the presence or absence of 0.1 μM C24:1 ceramide and/or 1 mM NAC for 3 days. (b) Intracellular ROS levels were measured using H_2_DCFDA (*n* = 3). (c) Myotubes were stained with anti‐MyHC antibody, and nuclei were counterstained with DAPI. Quantitative analyses of myogenic parameters per field are shown (*n* = 3). (d) Expression level of MyHC was assessed by western blotting under the same conditions (*n* = 3). (e,f) C2C12 cells were treated with 0.1 μM C24:1 ceramide and/or 1 mM NAC for 1 day (*n* = 3). (e) SA‐β‐gal‐positive cells (%) per field were quantified, and representative images of SA‐β‐gal staining are shown. SA‐β‐gal‐positive cells appear blue. (f) Western blot analyses were performed to assess p21 and p16 expression levels. β‐Tubulin served as a loading control. Scale bars: 100 μm (b and c), 20 μm (e). ROS, reactive oxygen species; NAC, N‐acetyl cysteine; MyHC, myosin heavy chain; DAPI, 4′,6‐diamidino‐2‐phenylindole; SA‐β‐gal, senescence‐associated β‐galactosidase. *p < 0.05 vs. untreated control; #*p* < 0.05 vs. 0.1 μM C24:1 ceramide.

C24:1 ceramide exposure also led to a dose‐dependent increase in intracellular ROS levels (Figure 4a), which was effectively neutralised by NAC pretreatment (Figure 4b). Co‐treatment with NAC not only attenuated ROS generation but also reversed the suppressive effects of C24:1 ceramide on myotube formation and MyHC protein expression (Figure 4c,d, respectively). In addition, similar to C18:0, C24:1 ceramide induced cellular senescence in C2C12 myoblasts, an effect that was also reversed by co‐treatment with the antioxidant NAC (Figure 4e,f). Taken together, these results underscore a shared oxidative mechanism underlying both the anti‐myogenic and pro‐senescent effects induced by C18:0 and C24:1 ceramides.

### 3.2 Impaired Muscle Phenotypes Following Systemic C18:0 and C24:1 Exposure in Mice

**FIGURE 5:**
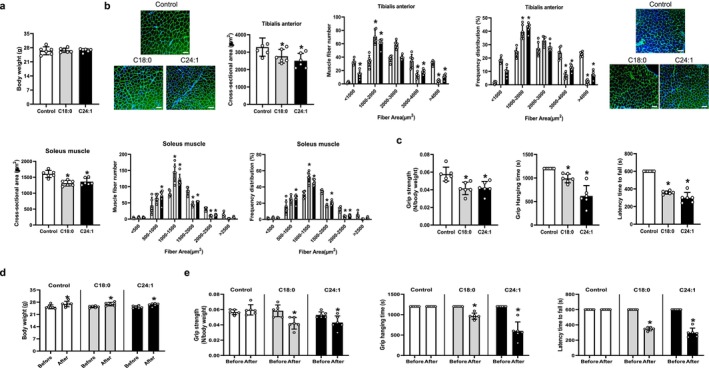
Systemic C18:0 and C24:1 ceramide treatment reduces muscle strength as well as the size of muscle fibre in mice. Three‐month‐old male mice were intraperitoneally injected with PBS (100 μL) or C18:0 or C24:1 ceramide (50 μg/100 μL) for 4 weeks (*n* = 6 per group). (a–c) Muscle phenotypes were compared between untreated controls and C18:0‐ or C24:1‐injected mice after 4 weeks of treatment. (a) Body weight; (b) size of the tibialis anterior and soleus muscles; and (c) grip strength, grid hanging time and latency to fall from the rotating rod. (d,e) Muscle phenotypes were also compared before and after the 4‐week treatment within each group. (d) Body weight; (e) grip strength, grid hanging time and latency to fall from the rotating rod. PBS, phosphate‐buffered saline. *p < 0.05 vs. untreated control or pre‐treatment values.

To evaluate the in vivo effects of C18:0 and C24:1 ceramides on skeletal muscle, 3‐month‐old male mice were administered daily intraperitoneal injections of 50 μg of either ceramide for 4 weeks. Control and treatment groups were matched for baseline body weight, and no significant differences in body weight were observed between groups at the end of the intervention (Figure 5a). Histological analysis revealed that both C18:0 and C24:1 ceramide treatments significantly reduced the cross‐sectional area (CSA) of the tibialis anterior (TA) muscle by 20.5% and 20.9%, respectively (Figure 5b). This reduction was accompanied by an increased proportion of small‐diameter myofibres and a corresponding decrease in large fibres (Figure 5b). Similar changes were observed in the soleus muscle, where CSA decreased by 18.1% with C18:0 ceramide and 16.1% with C24:1 ceramide, again with a shift toward smaller fibre sizes (Figure 5b). Consistent with the in vitro observations of ceramide‐induced inhibition of myogenesis and the in vivo reduction in muscle fibre CSA, DAPI counterstaining revealed no increase in centrally located nuclei (Figure S1), which are characteristic of regenerative fibres. Furthermore, immunostaining of both TA and soleus muscles revealed that C18:0 and C24:1 ceramide treatments did not alter the proportions of type I, type IIa or type IIb fibres, indicating that these ceramides do not induce muscle fibre‐type switching, but instead drive muscle atrophy (Figure S2). Functional assessments demonstrated significant impairments in muscle performance following ceramide exposure. Compared to controls, both C18:0‐ and C24:1‐treated mice exhibited reduced grip strength, shorter grid hanging times, and decreased latency to fall in the rota‐rod test (Figure 5c). Longitudinal analysis within each group showed that body weight increased similarly across all groups during the 4‐week period (Figure 5d). However, functional parameters declined significantly in both ceramide‐treated groups, whereas no notable changes were observed in untreated controls (Figure 5e).

### 3.3 Ceramides Induce Muscle Atrophy Through ROS‐Dependent Suppression of ITGB1 Signalling

**FIGURE 6:**
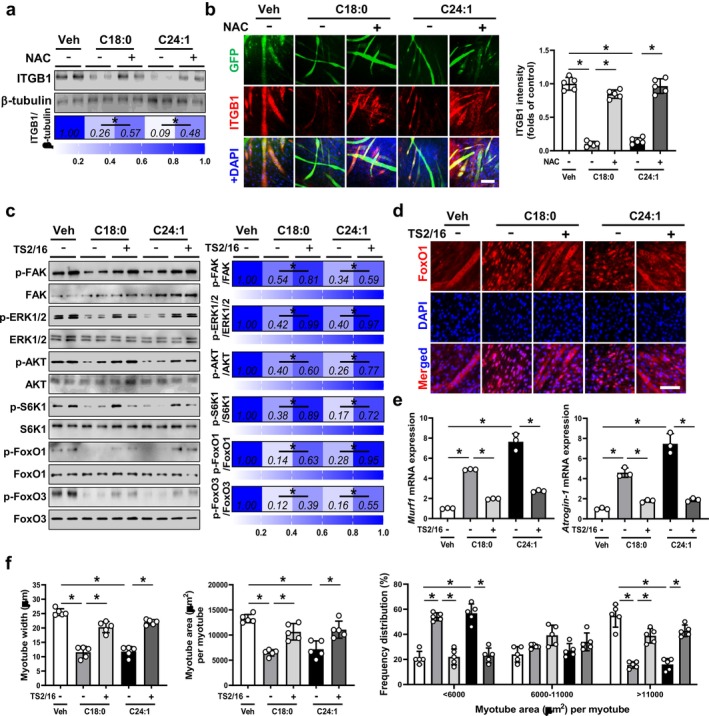
Ceramide‐induced oxidative stress suppresses ITGB1 signalling and promotes skeletal muscle atrophy in vitro. (a,b) Differentiated C2C12 myotubes were treated with vehicle (Veh), C18:0 ceramide or C24:1 ceramide in the presence or absence of NAC. (a) Western blot analyses were performed to assess ITGB1 protein expression (*n* = 3). (b) Myotubes were immunostained with an anti‐ITGB1 antibody, and nuclei were counterstained with DAPI (*n* = 5). (c) Western blot analyses were conducted to evaluate the activation status of ITGB1‐associated signalling pathways in myotubes treated with C18:0 or C24:1 ceramide in the presence or absence of the ITGB1‐activating antibody TS2/16. (d) Myotubes were immunostained with an anti‐FoxO1 antibody to assess nuclear localisation following treatment with C18:0 or C24:1 ceramide with or without TS2/16. Nuclei were counterstained with DAPI (*n* = 5). (e) mRNA expression levels of *MuRF1* and *Atrogin‐1* were evaluated by quantitative reverse transcription polymerase chain reaction (*n* = 3). (f) Myotube width, myotube area per myotube and myotube size distribution were quantified to assess myotube atrophy following ceramide treatment with or without TS2/16 (*n* = 5). Scale bars: 100 μm (b,d). An asterisk (*) indicates a statistically significant difference among groups (b–f).

To further delineate the molecular mechanisms linking ceramide‐induced oxidative stress to muscle atrophy, we focused on integrin β1 (ITGB1), a key regulator of muscle structural integrity and integrin‐mediated anabolic signalling [15]. In differentiated C2C12 myotubes, exposure to either C18:0 or C24:1 ceramide markedly reduced ITGB1 protein expression (Figure 6a). Notably, co‐treatment with NAC largely restored ITGB1 levels (Figure 6a), consistent with its ability to attenuate ceramide‐induced mitochondrial ROS (Figure S3a), suggesting that oxidative stress contributes to ITGB1 suppression. Immunocytochemical analyses further confirmed the reduction of ITGB1 abundance in ceramide‐treated myotubes and its recovery following NAC treatment (Figure 6b). Given the well‐established role of ITGB1 in coordinating FAK‐ERK‐AKT signalling [15, 16, 17, 18], we next examined the activation status of this pathway. Exposure to either C18:0 or C24:1 ceramide significantly reduced the phosphorylation levels of FAK, ERK1/2, AKT and S6K1 (Figure 6c), indicating the disruption of ITGB1‐dependent anabolic signalling. Concomitantly, phosphorylation of the transcription factors FoxO1 and FoxO3 was substantially reduced, suggesting the activation of FoxO‐dependent catabolic programs.

To further establish the functional relevance of ITGB1 signalling, myotubes were treated with the integrin β1‐activating antibody TS2/16. TS2/16 restored downstream signalling through the FAK‐AKT axis and counteracted ceramide‐induced signalling suppression (Figure 6c). Immunocytochemical analyses further demonstrated pronounced nuclear accumulation of FoxO1 in myotubes exposed to C18:0 or C24:1 ceramide (Figures 6d and S3b), which was accompanied by increased expression of the canonical muscle atrophy‐associated genes *MuRF1* and *Atrogin‐1* (Figure 6e). Conversely, restoration of ITGB1 signalling by TS2/16 mitigated ceramide‐induced myotube atrophy, as reflected by increased myotube width and area, alongside a shift in the size distribution toward larger fibres (Figure 6f).

To validate these findings in vivo, we next examined ITGB1 signalling in skeletal muscle tissues from ceramide‐treated mice. ITGB1 protein expression was significantly reduced in both tibialis anterior and soleus muscles following systemic administration of C18:0 or C24:1 ceramide (Figures S4a and S4b). Downstream signalling analyses in soleus muscle further confirmed suppression of the ITGB1‐FAK‐AKT signalling axis (Figure S4c). Nuclear protein extracts revealed increased nuclear accumulation of FoxO1 and FoxO3 (Figure S5), indicating activation of FoxO‐dependent transcriptional programs in vivo. In line with these signalling alterations, mRNA expression levels of *MuRF1* and *Atrogin‐1* were significantly elevated in the soleus muscle of ceramide‐treated mice (Figure S4d). Together, these findings indicate that ceramide‐induced oxidative stress suppresses ITGB1 signalling, thereby disrupting the FAK‐AKT anabolic signalling axis and promoting FoxO‐dependent muscle atrophy programmes (Figure S4e).

### 3.4 Association of Elevated Circulating C18:0 and C24:1 Ceramide Levels With Increased Risk of Sarcopenia in Older Adults

To assess the clinical relevance of the experimental findings, we analysed the association between circulating ceramide levels and muscle phenotypes in community‐dwelling older adults aged ≥ 65 years. Table S1 summarises baseline characteristics stratified by sarcopenia status. Compared with non‐sarcopenic individuals, those with sarcopenia were significantly older and had lower body weight and BMI (*p* < 0.001–0.004). As expected, measures of muscle quantity (appendicular skeletal muscle mass, skeletal muscle index), strength (grip strength) and function (gait speed, short physical performance battery, chair stand time) were all significantly impaired in the sarcopenia group (*p* < 0.001–0.003). No significant differences were observed in sex distribution, height, diabetes prevalence, polypharmacy or fall history. Notably, serum concentrations of both C18:0 and C24:1 ceramides were significantly higher in individuals with sarcopenia than in those without (both *p* = 0.002).

**FIGURE 7:**
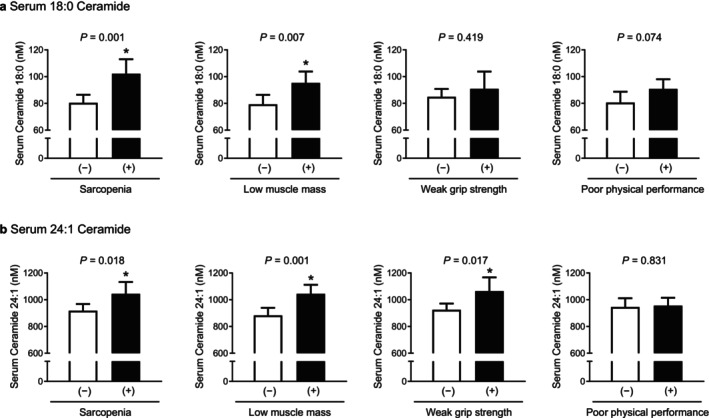
Differences in serum C18:0 (a) and C24:1 (b) ceramide levels based on sarcopenia status and the related parameters after adjusting for sex, age and BMI. The estimated means with 95% confidence intervals were generated and compared using analysis of covariance. An asterisk (*) indicates a statistically significant difference from the control.

ANCOVA analyses adjusted for sex, age and BMI revealed that participants with sarcopenia exhibited a 27% higher mean serum C18:0 ceramide level compared to those without (*p* = 0.001), and a 20% elevation was observed in participants with low muscle mass (*p* = 0.007) (Figure 7a). However, serum C18:0 ceramide did not differ significantly based on grip strength or physical performance. Conversely, serum C24:1 ceramide levels were significantly elevated in individuals with sarcopenia (14% increase), low muscle mass (18%) and weak grip strength (15%) compared to controls (*p* = 0.001–0.018), while no significant association was found with poor physical performance (Figure 7b).

Logistic regression analysis adjusted for sex, age and BMI demonstrated that each SD increase in serum C18:0 ceramide was associated with a 1.99‐fold higher odds of sarcopenia (95% CI 1.26–3.13, *p* = 0.003) and a 2.10‐fold higher odds of low muscle mass (95% CI 1.26–3.50, *p* = 0.005), but not with reduced strength or performance (Table 1). In contrast, elevated serum C24:1 ceramide was associated with increased odds of sarcopenia (OR 1.57, 95% CI 1.02–2.41, *p* = 0.040), low muscle mass (OR 2.38, 95% CI 1.43–3.95, *p* = 0.001) and weak grip strength (OR 1.58, 95% CI 1.02–2.43, *p* = 0.040), but not with poor physical performance.

**TABLE 1:** Association of presence of sarcopenia or abnormalities in sarcopenia parameters with serum C18:0 or C24:1 ceramide level by multiple logistic regression analysis after adjusting for sex, age and BMI.

Dependent variable	Serum C18:0 ceramide		Serum C24:1 ceramide	
	OR (95% CI) per serum SD increment	*p*	OR (95% CI) per serum SD increment	*p*
Sarcopenia	**1.99 (1.26–3.13)**	**0.003**	**1.57 (1.02–2.41)**	**0.040**
Low muscle mass	**2.10 (1.26–3.50)**	**0.005**	**2.38 (1.43–3.95)**	**0.001**
Weak grip strength	1.18 (0.77–1.81)	0.450	**1.58 (1.02–2.43)**	**0.040**
Poor physical performance	1.37 (0.97–1.94)	0.079	1.04 (0.73–1.48)	0.824

*Note:* Bold numbers indicate statistically significant values.

Abbreviations: BMI, body mass index; CI, confidence interval; OR, odds ratio; SD, standard deviation.

## 4 Discussion

While sarcopenia was historically regarded as an inevitable consequence of aging, emerging insights into muscle metabolism now indicate that age‐related muscle loss may be reversible, leading to a paradigm shift in which sarcopenia is viewed as a modifiable condition [19, 20]. Consequently, global efforts to identify risk factors and develop therapeutic strategies for sarcopenia have gained substantial momentum in our rapidly aging society [21]. This study presents a comprehensive mechanistic framework integrating in vitro, animal, and clinical evidence, highlighting circulating C18:0 and C24:1 ceramides as novel factors strongly associated with sarcopenia. Experimental models revealed that both ceramide species disrupt myogenesis through ROS‐mediated pathways, leading to impaired muscle cell differentiation. Rodent studies demonstrated that ceramide administration recapitulated key sarcopenic phenotypes, including reduced muscle fibre cross‐sectional area, diminished grip strength, and impaired physical performance metrics. Clinically, we identified significantly elevated serum levels of C18:0 and C24:1 ceramides in older adults with sarcopenia compared to age‐matched controls. These findings suggest that ceramides may serve not merely as circulating biomarkers of muscle deterioration, but, as supported by our experimental models, may also potentially contribute to the pathophysiological cascade underlying age‐related sarcopenia.

Our mechanistic data further refine this concept by identifying ITGB1 signalling as a critical downstream node linking ceramide‐induced oxidative stress to muscle atrophy. The observed elevation in intracellular ROS following ceramide exposure is consistent with previous reports that ceramides disrupt mitochondrial function, resulting in excessive ROS generation [22]. Elevated ROS levels are known to impair the differentiation capacity of myogenic precursor cells by interfering with key myogenic regulatory factors and signalling pathways essential for muscle regeneration [23, 24, 25]. Furthermore, oxidative stress can induce cellular senescence and apoptosis, thereby compromising the pool and function of satellite cells required for effective muscle repair [26, 27]. Beyond these anti‐myogenic effects, our findings suggest that ceramide‐triggered oxidative stress may converge on ITGB1 signalling, a pathway central to maintaining myofibre structural integrity and transducing extracellular matrix‐derived anabolic cues within skeletal muscle [16]. In the present study, both ceramides reduced ITGB1 expression in a ROS‐dependent manner, accompanied by the attenuation of downstream FAK‐ERK‐AKT signalling. Suppression of ERK and S6K phosphorylation is well established to restrain protein synthesis, whereas activation of downstream catabolic programs promotes proteolytic remodelling, collectively shifting muscle proteostasis toward net protein loss [28]. Disruption of integrin signalling is therefore poised not only to weaken structural support but also to disengage myofibres from growth‐promoting signalling networks required for the maintenance of muscle mass. In this context, ceramide‐induced ITGB1 repression represents a critical molecular switch through which oxidative stress is translated into a sustained catabolic state. Additionally, a key downstream consequence of this signalling perturbation is the activation of FoxO transcription factors, which constitute central regulators of skeletal muscle atrophy. Activation of FoxO signalling promotes the transcription of canonical atrogenes, including the E3 ubiquitin ligases *MuRF1* and *Atrogin‐1*, thereby driving ubiquitin‐proteasome‐mediated degradation of myofibrillar proteins during muscle wasting [29, 30]. Consistent with this framework, ceramide exposure in our models resulted in reduced phosphorylation and enhanced nuclear accumulation of FoxO1/3, together with the induction of atrophy‐related gene expression. These findings collectively position ceramides upstream of a signalling cascade linking oxidative stress, ITGB1 suppression, and FoxO‐driven catabolic remodelling, thereby providing a coherent mechanistic explanation for the pronounced atrophic phenotype observed in our experimental systems.

Our findings should also be interpreted within the broader context of sphingolipid metabolism in skeletal muscle. Emerging evidence indicates that enzymes regulating ceramide turnover are closely linked to muscle homeostasis and protein metabolism [31, 32, 33, 34]. Prior studies have shown that impairment of the ceramide kinase/ceramide‐1‐phosphate axis promotes skeletal muscle atrophy [31], whereas ceramide‐to‐sphingosine‐1‐phosphate conversion can support adaptive muscle responses [32]. In addition, acid ceramidase‐related abnormalities provide further evidence that disruption of sphingolipid flux can contribute to pathological ceramide accumulation [33, 34]. Together, these observations support the concept that balanced sphingolipid metabolism is essential for skeletal muscle integrity. Our data extend this framework by showing that two specific circulating ceramide species, C18:0 and C24:1, impair myogenesis and promote atrophic remodelling in experimental models and are associated with sarcopenia in older adults.

An additional point of interest is the differential clinical pattern observed between the two ceramide species. Although both C18:0 and C24:1 ceramides were associated with low muscle mass, only C24:1 showed a significant association with weak grip strength in older adults. One possible explanation is that the two ceramides capture partially distinct aspects of muscle pathology. Reduced muscle mass primarily reflects muscle quantity, whereas muscle strength is additionally influenced by muscle quality, contractile efficiency, neuromuscular activation, and other integrative determinants [7]. In this context, the broader association of C24:1 with both low muscle mass and weak grip strength may indicate that it reflects a wider spectrum of adverse muscle phenotypes in humans. Notably, although C18:0 ceramide reduced grip strength in the mouse model, its association with weak grip strength was not statistically significant in the clinical cohort. This discordance may reflect differences between experimental and clinical contexts, including species differences, direct short‐term exogenous ceramide exposure in young mice versus chronic endogenous exposure in heterogeneous older adults, and the more complex determinants of muscle strength in humans. Nevertheless, because our clinical analysis was cross‐sectional and the sample size was modest, these phenotype‐specific differences require confirmation in larger independent cohorts.

From a clinical standpoint, our findings carry several important implications for the prevention and management of sarcopenia. First, circulating ceramide species—specifically C18:0 and C24:1—may serve as readily measurable, minimally invasive biomarkers for identifying older adults at heightened risk for sarcopenia. Given the progressive and often subclinical nature of muscle loss in its early stages, the ability to stratify individuals based on serum ceramide levels could enable timely intervention before functional decline becomes irreversible. Second, our results highlight ceramide metabolism as a promising therapeutic target. Pharmacologic inhibitors of key enzymes in the ceramide biosynthesis pathway, such as serine palmitoyltransferase or ceramide synthases, have demonstrated efficacy in reducing ceramide accumulation and improving metabolic outcomes in preclinical models [35, 36, 37]. These agents could potentially be repurposed or optimised for muscle‐preserving therapies in aging populations. Furthermore, non‐pharmacologic approaches that influence ceramide homeostasis warrant consideration. Regular physical activity has been shown to modulate lipid metabolism and reduce ceramide burden, in part by enhancing mitochondrial function and lowering oxidative stress [38, 39]. Similarly, dietary interventions—such as reduced intake of saturated fats or supplementation with polyunsaturated fatty acids—may alter ceramide synthesis and turnover [40]. Taken together, our data suggest that both pharmacologic and lifestyle‐based strategies targeting ceramide accumulation could represent viable avenues for attenuating age‐related muscle decline.

Despite the promising nature of our findings, several limitations must be acknowledged. First, the cross‐sectional nature of the clinical component limits causal inference between elevated ceramide levels and sarcopenia development. While our in vitro and in vivo data provide strong biological plausibility for a pathogenic role of ceramides, longitudinal studies are needed to determine whether elevated ceramide levels precede and predict subsequent muscle deterioration in aging populations. Second, muscle mass was assessed using BIA rather than dual‐energy X‐ray absorptiometry (DXA), which is the gold standard for body composition analysis. Although BIA is a highly accessible and validated tool for community‐based sarcopenia assessments according to the AWGS guidelines, its precision in quantifying appendicular skeletal muscle mass may be relatively lower than that of DXA. Third, our study was conducted exclusively among community‐dwelling older adults of Korean ethnicity. While this minimises heterogeneity due to ethnic and socioeconomic factors, it may also limit the external validity of our findings. Fourth, we cannot rule out the possibility that our clinical findings could be influenced by unmeasured factors that may affect circulating ceramide levels and/or muscle health in humans, such as dietary fat intake, physical activity level and lipid‐lowering medication use. Finally, the in vivo experiments were performed in healthy male C57BL/6 mice to test whether systemic ceramide exposure is sufficient to induce sarcopenia‐like muscle phenotypes. Thus, this model may not fully reflect the physiological complexity of age‐related sarcopenia. Validation in aged or accelerated‐aging models, such as SAMP10 mice, would further strengthen the translational relevance of our findings.

In conclusion, our study provides integrative evidence from cellular, animal, and clinical settings that implicates the C18:0 and C24:1 ceramides as potential mediators of sarcopenia. These ceramides impaired myogenesis via oxidative stress and induced reductions in muscle mass, strength, and physical performance in mice. Mechanistically, ceramide‐induced oxidative stress suppressed ITGB1‐dependent anabolic signalling and activated FoxO‐associated catabolic programs, thereby promoting skeletal muscle atrophy. Clinically, elevated circulating levels of C18:0 and C24:1 ceramides were significantly associated with sarcopenia in older adults. Together, these findings support a mechanistic role for ceramides in muscle deterioration and highlight their potential as biomarkers and therapeutic targets for age‐related muscle decline.

## Funding

This research was supported by grants from the Korean ARPA‐H Project through the Korea Health Industry Development Institute (KHIDI), funded by the Ministry of Health and Welfare, Republic of Korea (Grant No. RS‐2024‐00507256), and the Korea Health Technology R&D Project through KHIDI, also funded by the Ministry of Health and Welfare, Republic of Korea (Grant Nos. RS‐2024‐00401934 and RS‐2025‐25459589).

## Conflicts of Interest

The authors declare no conflicts of interest.

## Supporting information


**Data S1:** Supplementary information.

**Table S1:** Basic clinical characteristics of the study participants.
**Figure S1:** Myonuclear localisation in skeletal muscle following systemic ceramide administration. Representative immunofluorescence images of the tibialis anterior and soleus muscles from mice treated with PBS (Control), C18:0 or C24:1 ceramide for 4 weeks. Sections were stained for laminin (green) to outline myofibres and counterstained with DAPI (blue) to visualise nuclei. In all groups, myonuclei are located at the periphery of the muscle fibres, with no evidence of centrally nucleated fibres. The bottom panels are magnified views of the boxed areas in the top panels. Scale bars: 100 μm (top) and 20 μm (bottom).
**Figure S2:** C18:0 and C24:1 ceramide treatments do not alter the MyHC fibre‐type composition in the tibialis anterior and soleus muscles. Three‐month‐old male mice were intraperitoneally injected with PBS (100 μL), C18:0 (50 μg/100 μL) or C24:1 ceramide (50 μg/100 μL) for 4 weeks (n = 6 per group). Representative immunofluorescence images of laminin (green) and MyHC I, MyHC IIa and MyHC IIb (red) are shown. The MyHC fibre type composition in tibialis anterior and soleus muscles was quantified. Scale bars: 100 μm. PBS, phosphate‐buffered saline; MyHC, myosin heavy chain.
**Figure S3:** C18:0 and C24:1 ceramides increase mitochondrial ROS and promote FoxO1 nuclear translocation in myotubes. (a) Mitochondrial ROS levels were assessed using MitoSOX staining in differentiated C2C12 myotubes treated with vehicle (Veh), C18:0 ceramide or C24:1 ceramide in the presence or absence of 1 mM NAC (n = 6). (b) Myotubes were immunostained with an anti‐FoxO1 antibody to evaluate nuclear localisation under the conditions indicated in (A). Quantitative analyses per field are shown (n = 5). An asterisk (*) indicates a statistically significant difference among groups.
**Figure S4:** Systemic ceramide exposure suppresses ITGB1 signalling and promotes muscle atrophy programs in vivo. (a,b) Western blot analyses were performed to assess ITGB1 protein expression in (a) tibialis anterior and (b) soleus muscles of mice treated with C18:0 or C24:1 ceramide (n = 3). (c) Western blot analyses were conducted to evaluate ITGB1‐associated signalling pathways in soleus muscle tissues (n = 3). (d) mRNA expression levels of MuRF1 and Atrogin‐1 in skeletal muscle tissues were assessed by quantitative reverse transcription polymerase chain reaction (n = 3). (e) Proposed model illustrating the mechanism. Created with BioRender. Wei, S. (2026) https://BioRender.com/k5k2e7c. An asterisk (*) indicates a statistically significant difference vs. untreated control (a–d).
**Figure S5:** C18:0 and C24:1 ceramides promote the nuclear accumulation of FoxO transcription factors in skeletal muscle. (a) Nuclear proteins were isolated from the skeletal muscle tissues of control and ceramide‐treated mice. Western blot analyses were performed to assess β‐tubulin and Histone H3 levels in the nuclear and cytosolic fractions. (b) Western blot analyses were performed to assess FoxO1 and FoxO3 levels in the nuclear fractions. Histone H3 served as a nuclear loading control (n = 3). *p < 0.05 vs. untreated control.
