# Supplementary material for: Elevated Circulating Ceramides 18:0 and 24:1 as a Risk Factor for Sarcopenia: In Vitro, Animal, and Clinical Evidence
Source: J Cachexia Sarcopenia Muscle. 2026 May 1;17(3):e70310. doi: 10.1002/jcsm.70310 (PMC13133597; doi:10.1002/jcsm.70310)
Supplement: Supplementary file 1 — Data S1: Supplementary information. [file JCSM-17-e70310-s002.docx]

**Supplementary Materials and Methods**

*Immunofluorescence*

Following differentiation, C2C12 cells were fixed in 4% paraformaldehyde (PFA) for 15 minutes at room temperature and rinsed twice with phosphate-buffered saline (PBS). Permeabilization was carried out using a buffer containing 0.01 M sodium citrate and 0.1% Triton X-100 for 10 minutes. After an additional two PBS washes, cells were incubated with 2% bovine serum albumin (BSA) in PBS for 1 hour at room temperature to block nonspecific binding sites. Cells were then incubated overnight at 4°C with a primary antibody targeting myosin heavy chain (MyHC; MF20, Developmental Studies Hybridoma Bank, Iowa City, IA). The following day, Alexa Fluor 555-conjugated secondary antibodies (1:1000 dilution; Cell Signaling Technology) were applied for 1 hour at room temperature. After washing with PBST (PBS containing 0.2% Tween-20), nuclear staining was performed using 4’,6-diamidino-2-phenylindole (DAPI, 1:10,000 dilution; Sigma-Aldrich, St. Louis, MO) for 2 minutes. Slides were mounted using Fluoromount G (Southern Biotech, Birmingham, AL), and fluorescence images were acquired using a Carl Zeiss fluorescence microscope (Jena, Germany). Myotubes were defined as MyHC-positive structures containing three or more nuclei within a shared cytoplasm. Myotube area was quantified using ZEN 2 (blue edition) software (Carl Zeiss), and the fusion index was calculated as: Fusion Index (%) = 100 × (number of nuclei in MyHC+ myotubes) / (total number of nuclei in MyHC+ myotubes and myocytes) [S1]. For immunofluorescence analysis of signaling proteins, differentiated myotubes were processed as described above and incubated overnight at 4°C with primary antibodies against ITGB1 (14-0299-82, Invitrogen), FoxO1 (MA5-14846, Invitrogen), or FoxO3 (MA5-14932, Invitrogen). After washing, cells were incubated with an Alexa Fluor 546-conjugated secondary antibody (1:400; Cell Signaling Technology) for 1 hour at room temperature. Fluorescence images were acquired using a Cytation 5 confocal microscopy system (Agilent), and fluorescence intensity was quantified using ImageJ software (National Institutes of Health, Bethesda, MD, USA).

*Western Blot Analysis*

Cells were lysed in radioimmunoprecipitation assay (RIPA) buffer containing 50 mM Tris-HCl (pH 7.4), 150 mM NaCl, 1% Triton X-100, 1 mM EDTA, 1 mM EGTA, 0.1% SDS, 1% sodium deoxycholate, 1 mM sodium orthovanadate (Na₃VO₄), 1 mM sodium fluoride (NaF), 1 mM phenylmethylsulfonyl fluoride (PMSF), and a protease inhibitor cocktail. After incubation on ice for 30 minutes, the lysates were clarified by centrifugation at 14,000 rpm for 20 minutes at 4°C. Nuclear protein fractions were prepared as previously described [S1]. Protein concentrations were determined using a BCA protein assay kit (Pierce, Rockford, IL). Equal amounts of protein were separated via SDS-PAGE and transferred onto polyvinylidene fluoride (PVDF) membranes. Membranes were then immunoblotted with primary antibodies against MyHC (MF20; Developmental Studies Hybridoma Bank) and myogenin (sc-12732; Santa Cruz Biotechnology, Dallas, TX), ITGB1 (14-0299-82, Invitrogen), β-tubulin (T8328, Sigma-Aldrich), p-ERK1/2 (SAB4301578, Sigma-Aldrich), ERK1/2 (SAB1305560, Sigma-Aldrich), p-AKT (SAB5703038, Sigma-Aldrich), AKT (SAB4500797, Sigma-Aldrich), p-FAK (700255, Invitrogen), FAK (AHO1272, Invitrogen), p-S6K1 (MA5-15202, Invitrogen), S6K1 (9202, Cell Signaling), p-FoxO1 (9461, Cell Signaling), FoxO1 (MA5-14846, Invitrogen), p-FoxO3 (13129, Cell Signaling), FoxO3 (MA5-14932, Invitrogen), and Histone H3 (ab1791, Abcam), followed by appropriate secondary antibodies.

*Quantitative Reverse-Transcription Polymerase Chain Reaction*

Total RNA was isolated using TRIzol reagent (Invitrogen, Carlsbad, CA) in accordance with the manufacturer’s instructions. Complementary DNA (cDNA) was synthesized from 1 µg of total RNA using the SuperScript III First-Strand Synthesis System (Invitrogen) with oligo(dT) primers. Quantitative PCR was performed in triplicate using the LightCycler® 480 SYBR Green I Master Mix (Roche, Mannheim, Germany). Primers specific for *myogenin* (NM_031189.2), *MyHC* (NM_001013397.2), *MuRF1* (Trim63; NM_001039048.2), and *Atrogin-1* (Fbxo32; NM_026346.3) were obtained from Applied Biosystems (Foster City, CA). Gene expression levels were normalized to 18S rRNA (NR_003278.3) using the ΔCt method.

*Migration Assay*

Cell migration was assessed using a Boyden chamber system equipped with transwell inserts containing an 8-μm pore size polycarbonate membrane (Corning, NY). C2C12 cells were seeded in the upper chamber at a density of 8 × 10⁴ cells per well in DMEM supplemented with 0.2% FBS. Synthetic ceramides were added to the lower chamber as a chemoattractant, and the cells were incubated for 5 hours. Following incubation, non-migrated cells on the upper surface of the membrane were removed with a cotton swab. Migrated cells on the lower membrane surface were fixed with 4% paraformaldehyde and stained with crystal violet. Stained cells were imaged using the cellSens Standard BX53 software (Olympus, Tokyo, Japan), and quantification was performed with ImageJ software (NIH, Bethesda, MD).

*Viability Assay*

Cell viability was evaluated using the Cell Counting Kit-8 (CCK-8; Dojindo, Kumamoto, Japan) according to the manufacturer’s protocol. Briefly, 10 μL of WST-8 reagent [2-(2-methoxy-4-nitrophenyl)-3-(4-nitrophenyl)-5-(2,4-disulfophenyl)-2H-tetrazolium, monosodium salt] was added to each well of a 96-well plate. After 1 hour of incubation, absorbance was measured at 450 nm with a reference wavelength of 650 nm using a microplate reader (SPECTRAmax 340PC; Molecular Devices, Palo Alto, CA).

*Measurement of Intracellular Reactive Oxygen Species Levels*

Intracellular reactive oxygen species (ROS) levels were assessed using the chloromethyl derivative of 2′,7′-dichlorofluorescein diacetate (CM-H₂DCFDA; C6827, Invitrogen). Cells were first rinsed with serum-free DMEM and then incubated with 10 μM CM-H₂DCFDA at 37°C for 30 minutes in the dark under 5% CO₂. After incubation, cells were washed with PBS and imaged using a fluorescence microscope (Carl Zeiss, Jena, Germany). Fluorescence intensity was also quantified with a microplate reader (Infinite 200PRO; Tecan Life Sciences, Zürich, Switzerland) at excitation/emission wavelengths of 490/520 nm, respectively.

*Senescence-Associated β-Galactosidase Staining*

The senescence-associated β-galactosidase (SA-β-Gal) assay was performed using a commercial kit (#9860; Cell Signaling Technology) according to the manufacturer’s instructions. Cells were seeded in 12-well plates and cultured under the indicated conditions for 24 h. After removing the culture medium, cells were rinsed once with PBS, fixed with the supplied fixative for 15 min at room temperature, and washed twice with PBS. A freshly prepared SA-β-Gal staining solution (pH 6.0) was then added, and the plates were incubated for 48 h at 37 °C in a dry, CO₂-free incubator. Following staining, SA-β-Gal–positive (blue) cells were visualized and imaged under a bright-field microscope (Olympus; total magnification, 200×).

*Primary Myoblast Isolation and Myogenic Differentiation*

Primary myoblasts were isolated from the hindlimb muscles of 2- to 3-week-old C57BL/6 mice. Muscles were dissected under sterile conditions, rinsed in PBS, and minced with sterile scissors. The tissue fragments were digested with Collagenase/Dispase (Roche, Mannheim, Germany) at 37 °C for 30 minutes, with gentle trituration every 10 minutes to enhance dissociation. The resulting suspension was passed through a 70 μm cell strainer and centrifuged at 500 × g for 5 minutes. Cells were pre-plated on uncoated dishes at 37 °C for 40 minutes to allow fibroblast attachment, and the non-adherent fraction was collected and resuspended in Ham’s F-10 growth medium containing 20% fetal bovine serum and 4% penicillin-streptomycin. To further enrich for myoblasts, cells were transferred to collagen-coated plates and cultured in Ham’s F-10 medium supplemented with 5 ng/mL recombinant human basic fibroblast growth factor (FGF-basic; Invitrogen, Carlsbad, CA). The culture medium was replaced every other day. Upon reaching approximately 90% confluence, myogenic differentiation was induced by switching to Dulbecco’s Modified Eagle’s Medium (DMEM) supplemented with 2% horse serum and 1% penicillin-streptomycin. Multinucleated myotubes typically appeared within 3 days of differentiation.

*Immunofluorescence and Muscle Fiber Size Measurement*

Frozen muscle tissues embedded in optimal cutting temperature (OCT) compound were sectioned at a thickness of 10 μm using a cryostat microtome (Leica Microsystems, Wetzlar, Germany). For cross-sectional area (CSA) analysis, the sections were subjected to immunofluorescent staining with laminin and DAPI. Briefly, tissue sections were blocked for 1 hour at room temperature and then incubated overnight at 4 °C with a primary anti-laminin antibody (1:1000; Sigma-Aldrich). After PBS washes, the sections were incubated for 1 hour with a secondary antibody (Alexa Fluor 647-conjugated goat anti-rabbit IgG, 1:1000; Invitrogen). Nuclei were counterstained with DAPI using an aqueous fluoroshield mounting medium. The CSA of individual muscle fibers was measured by manually tracing the laminin-positive outlines using ZEN 2 (blue edition) software (Carl Zeiss).

*Skeletal Muscle Function Exploration in Mice*

Skeletal muscle performance was evaluated using a series of functional tests. Forelimb grip strength was measured by allowing the mouse to grasp a metal grid with all four limbs while gently pulling it backward. This procedure was repeated five times per mouse; the lowest value was excluded, and the mean of the remaining four measurements was calculated and reported in Newtons (N). Muscle strength was further assessed using Kondziella’s inverted screen test. To enhance the sensitivity of this assay, a small weight equivalent to 8–10% of the mouse's body weight was affixed to the tail, as previously described [S2]. Mice were then placed on a wire mesh screen, which was gradually inverted, and the latency to fall was recorded as an index of muscle strength. Motor coordination and endurance were evaluated using a rota-rod apparatus (Jeungdo Bio & Plant Co., Seoul, South Korea). Each mouse was positioned on a rotating rod, and the latency to fall as well as the distance traveled prior to falling were measured. To ensure consistent performance, mice were familiarized with the testing apparatus through acclimation sessions conducted one day prior to the experiment. All tests were performed twice per animal with a resting interval between trials to ensure measurement reliability.

*Assessment of Sarcopenia in Older Adults*

Trained research nurses obtained demographic and clinical information through structured interviews and review of electronic medical records. Body composition, including appendicular skeletal muscle mass (ASM), was evaluated using multifrequency bioelectrical impedance analysis (BIA) with the InBody S10 device (InBody, Seoul, Korea), operating at 1, 5, 50, 250, 500, and 1,000 kHz [S3]. ASM was defined as the sum of lean mass in the arms and legs, and the skeletal muscle mass index (SMI) was calculated by dividing ASM by height squared (kg/m²). Muscle strength was assessed via handgrip strength in the dominant hand using a digital dynamometer (Patterson Medical, Warrenville, IL, USA). Participants were seated with the elbow flexed at 90°, and instructed to perform maximal isometric contraction. Two trials were conducted with a 1-minute interval, and the higher value was used for analysis. Physical performance was measured through gait speed over a 4-meter course, the time to complete five consecutive chair stands, and the short physical performance battery (SPPB), which comprises balance, gait speed, and chair stand tests [S4].

Sarcopenia, which is classified under the ICD-10 code M62.84, was defined according to the 2019 consensus of the Asian Working Group for Sarcopenia (AWGS) [S5], requiring the presence of low muscle mass with either weak muscle strength or poor physical performance. Low muscle mass was defined as SMI <7.0 kg/m² for men and <5.7 kg/m² for women. Weak muscle strength was defined as handgrip strength <28 kg for men and <18 kg for women. Poor physical performance was defined as gait speed <1.0 m/s, chair stand time >12 seconds, or an SPPB score ≤9.

*Measurement of Ceramides in Human Serum*

After an overnight fast of ≥8 hours, venous blood samples were collected from the antecubital vein during morning hours. Samples were immediately centrifuged at 3,000 rpm for 5 minutes at 4°C, and the supernatant was collected to remove cellular debris. Specimens showing clotting or hemolysis were excluded. An internal standard solution containing 500 nM C17 ceramide (Avanti Polar Lipids, Alabaster, AL, USA) was added to the serum prior to lipid extraction. Sphingolipids were extracted using the Bligh and Dyer method [S6]. Ceramides were quantified via liquid chromatography–tandem mass spectrometry (LC-MS/MS) using a 1290 HPLC system (Agilent Technologies, Santa Clara, CA, USA) coupled with a QTRAP 5500 mass spectrometer (AB Sciex, Framingham, MA, USA). Analyses were performed in positive ion mode with multiple reaction monitoring (MRM), and quantification was based on extracted ion chromatograms of targeted lipid transitions. Calibration curves ranged from 0.1 to 1,000 nmol/L, with correlation coefficients (r) ≥0.99. Data were processed using Analyst 1.5.2 (AB Sciex) or Xcalibur (Thermo Fisher Scientific, Waltham, MA, USA).

**Supplementary References**

S1. Wei S, Park SJ, Choi E, Jang IY, Zhang Y, Xue Y, et al. Detrimental Effects of β2-Microglobulin on Muscle Metabolism: Evidence From In Vitro, Animal and Human Research. J Cachexia Sarcopenia Muscle. 2025;16:e13745.

S2. Kang JS, Kim D, Rhee J, Seo JY, Park I, Kim JH, et al. Baf155 regulates skeletal muscle metabolism via HIF-1a signaling. PLoS Biol. 2023;21:e3002192.

S3. Oh JH, Song S, Rhee H, Lee SH, Kim DY, Choe JC, et al. Normal Reference Plots for the Bioelectrical Impedance Vector in Healthy Korean Adults. J Korean Med Sci. 2019;34:e198.

S4. Jung HW, Roh H, Cho Y, Jeong J, Shin YS, Lim JY, et al. Validation of a Multi-Sensor-Based Kiosk for Short Physical Performance Battery. J Am Geriatr Soc. 2019;67:2605-9.

S5. Chen LK, Woo J, Assantachai P, Auyeung TW, Chou MY, Iijima K, et al. Asian Working Group for Sarcopenia: 2019 Consensus Update on Sarcopenia Diagnosis and Treatment. J Am Med Dir Assoc. 2020;21:300-7.e2.

S6. Bligh EG, Dyer WJ. A rapid method of total lipid extraction and purification. Can J Biochem Physiol. 1959;37:911-7.
