# Supplementary material for: Elevated Circulating Ceramides 18:0 and 24:1 as a Risk Factor for Sarcopenia: In Vitro, Animal, and Clinical Evidence
Source: J Cachexia Sarcopenia Muscle. 2026 May 1;17(3):e70310. doi: 10.1002/jcsm.70310 (PMC13133597; doi:10.1002/jcsm.70310)
Supplement: Supplementary file 2 — Table S1: Basic clinical characteristics of the study participants. Figure S1: Myonuclear localisation in skeletal muscle following systemic ceramide administration. Representative immunofluorescence images of the tibialis anterior and soleus muscles from mice treated with PBS (Control), C18:0 or C24:1 ceramide for 4 weeks. Sections were stained for laminin (green) to outline myofibres and counterstained with DAPI (blue) to visualise nuclei. In all groups, myonuclei are located at the periphery of the muscle fibres, with no evidence of centrally nucleated fibres. The bottom panels are magnified views of the boxed areas in the top panels. Scale bars: 100 μm (top) and 20 μm (bottom). Figure S2: C18:0 and C24:1 ceramide treatments do not alter the MyHC fibre‐type composition in the tibialis anterior and soleus muscles. Three‐month‐old male mice were intraperitoneally injected with PBS (100 μL), C18:0 (50 μg/100 μL) or C24:1 ceramide (50 μg/100 μL) for 4 weeks (n = 6 per group). Representative immunofluorescence images of laminin (green) and MyHC I, MyHC IIa and MyHC IIb (red) are shown. The MyHC fibre type composition in tibialis anterior and soleus muscles was quantified. Scale bars: 100 μm. PBS, phosphate‐buffered saline; MyHC, myosin heavy chain. Figure S3: C18:0 and C24:1 ceramides increase mitochondrial ROS and promote FoxO1 nuclear translocation in myotubes. (a) Mitochondrial ROS levels were assessed using MitoSOX staining in differentiated C2C12 myotubes treated with vehicle (Veh), C18:0 ceramide or C24:1 ceramide in the presence or absence of 1 mM NAC (n = 6). (b) Myotubes were immunostained with an anti‐FoxO1 antibody to evaluate nuclear localisation under the conditions indicated in (A). Quantitative analyses per field are shown (n = 5). An asterisk (*) indicates a statistically significant difference among groups. Figure S4: Systemic ceramide exposure suppresses ITGB1 signalling and promotes muscle atrophy programs in vivo. (a,b) Western blot analyses were p [file JCSM-17-e70310-s001.docx]

**Supplementary Table 1.** Basic clinical characteristics of the study participants

|  | No sarcopenia  (n = 123) | Sarcopenia  (n = 42) | *p* |
| --- | --- | --- | --- |
| Age, y | **75.2 ± 5.2** | **79.7 ± 4.8** | **<0.001** |
| Female | 102 (82.9) | 31 (73.8) | 0.257 |
| Body weight, kg | **59.6 ± 9.5** | **53.5 ± 5.6** | **<0.001** |
| Height, cm | 154.9 ± 6.5 | 153.0 ± 6.8 | 0.113 |
| Body mass index, kg/m^2^ | **25.4 ± 5.4** | **22.9 ± 2.5** | **0.004** |
| Diabetes mellitus | 48 (39.0) | 14 (33.3) | 0.582 |
| Polypharmacy | 63 (51.2) | 26 (61.9) | 0.283 |
| Fall in previous year | 22 (17.9) | 8 (19.0) | 0.821 |
| Appendicular skeletal muscle mass, kg | **15.2 ± 2.9** | **13.1 ± 2.2** | **<0.001** |
| Skeletal muscle mass index, kg/m^2^ | **6.31 ± 0.79** | **5.55 ± 0.52** | **<0.001** |
| Grip strength, kg | **25.9 ± 6.2** | **19.8 ± 5.0** | **<0.001** |
| Usual gait speed, m/s | **1.04 ± 0.23** | **0.78 ± 0.26** | **<0.001** |
| Chair stand test time, s | **10.5 ± 5.4** | **17.0 ± 13.2** | **0.003** |
| SPPB total score (ranges, 0-12) | **10.9 ± 1.6** | **8.3 ± 3.1** | **<0.001** |
| SPS score (range, 0-3) | **0.72 ± 0.56** | **2.38 ± 0.49** | **<0.001** |
| Serum C18:0 ceramide, nM | **80.0 ± 29.2** | **103.4 ± 43.4** | **0.002** |
| Serum C24:1 ceramide, nM | **899.7 ± 237.6** | **1098.5 ± 379.5** | **0.002** |

Data are presented as mean ± standard deviation or n (%). Differences between the two groups were assessed using Student’s *t-*tests for continuous variables and χ^2^ test for categorical variables. Bold numbers indicate statistically significant values. SPPB, short physical performance battery; SPS, sarcopenia phenotype score.

**
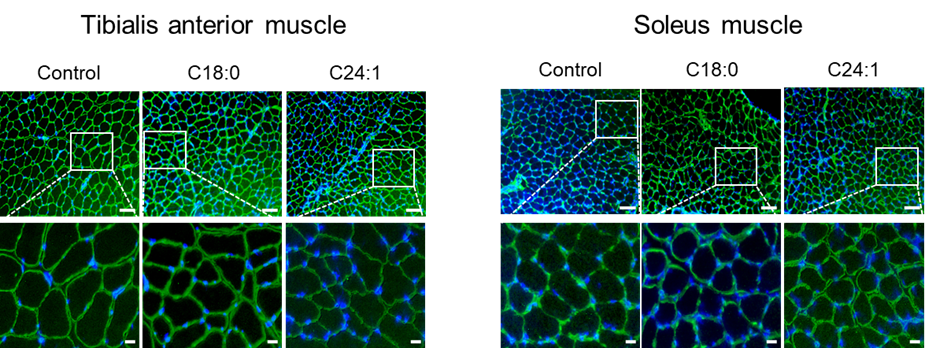
**

**Supplementary Figure 1.** Myonuclear localization in skeletal muscle following systemic ceramide administration. Representative immunofluorescence images of the tibialis anterior and soleus muscles from mice treated with PBS (Control), C18:0, or C24:1 ceramide for 4 weeks. Sections were stained for laminin (green) to outline myofibers and counterstained with DAPI (blue) to visualize nuclei. In all groups, myonuclei are located at the periphery of the muscle fibers, with no evidence of centrally nucleated fibers. The bottom panels are magnified views of the boxed areas in the top panels. Scale bars: 100 μm (top) and 20 μm (bottom).


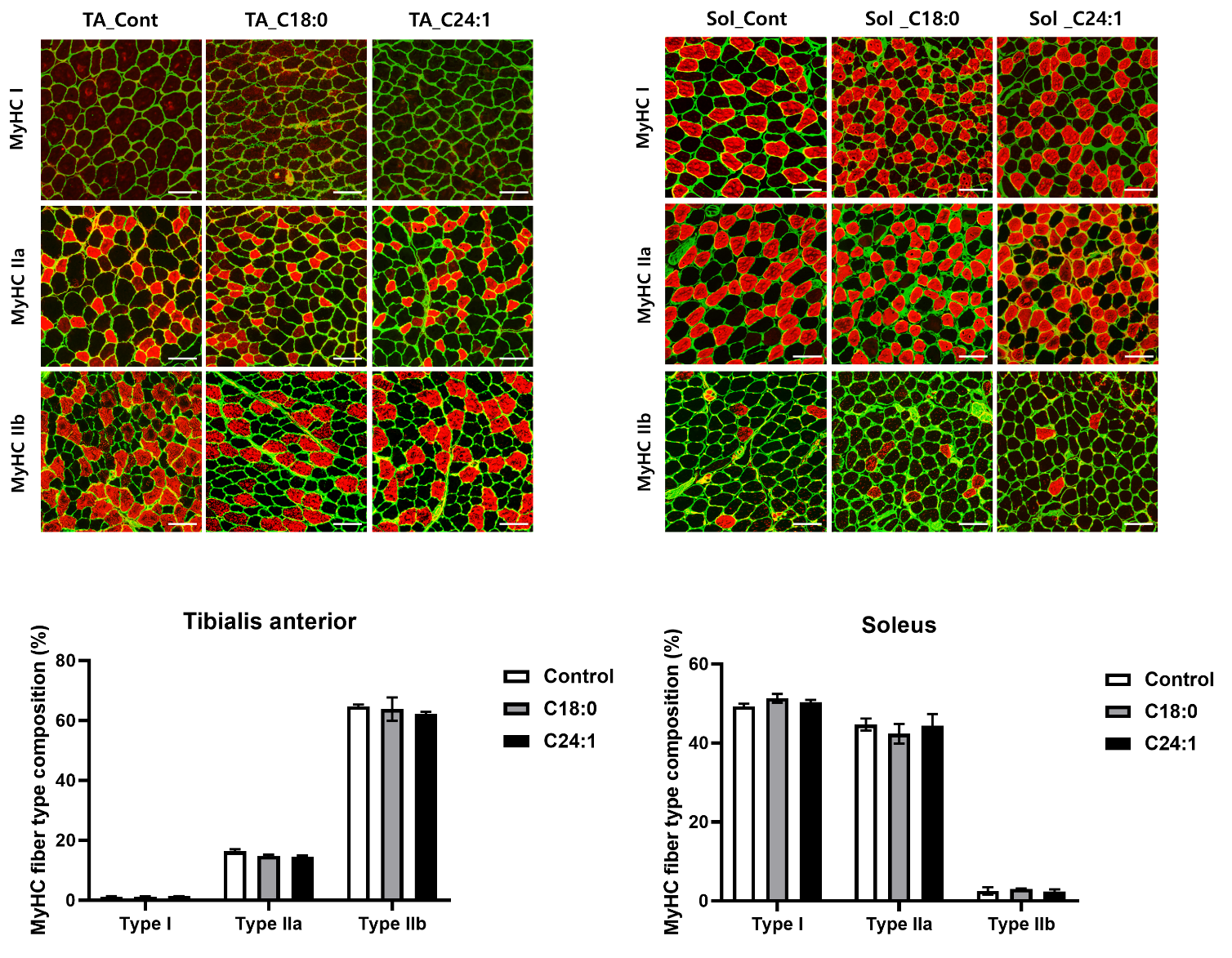


**Supplementary Figure 2.** C18:0 and C24:1 ceramide treatments do not alter the MyHC fiber-type composition in the tibialis anterior and soleus muscles. Three-month-old male mice were intraperitoneally injected with PBS (100 μL), C18:0 (50 μg/100 μL) or C24:1 ceramide (50 μg/100 μL) for 4 weeks (n = 6 per group). Representative immunofluorescence images of laminin (green) and MyHC I, MyHC IIa, and MyHC IIb (red) are shown. The MyHC fiber type composition in tibialis anterior and soleus muscles was quantified. Scale bars: 100 μm. PBS, phosphate-buffered saline; MyHC, myosin heavy chain.

**
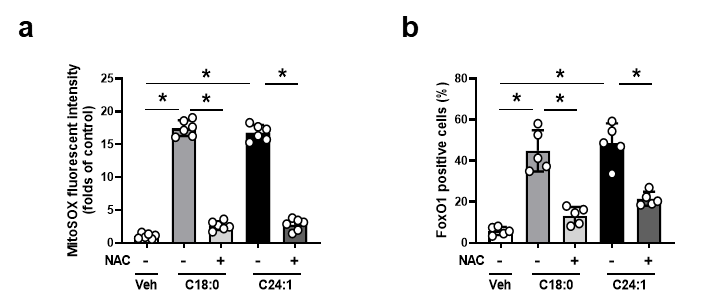
**

**Supplementary Figure 3.** C18:0 and C24:1 ceramides increase mitochondrial ROS and promote FoxO1 nuclear translocation in myotubes. (a) Mitochondrial ROS levels were assessed using MitoSOX staining in differentiated C2C12 myotubes treated with vehicle (Veh), C18:0 ceramide, or C24:1 ceramide in the presence or absence of 1 mM NAC (n = 6). (b) Myotubes were immunostained with an anti-FoxO1 antibody to evaluate nuclear localization under the conditions indicated in (A). Quantitative analyses per field are shown (n = 5). An asterisk (*) indicates a statistically significant difference among groups.

**
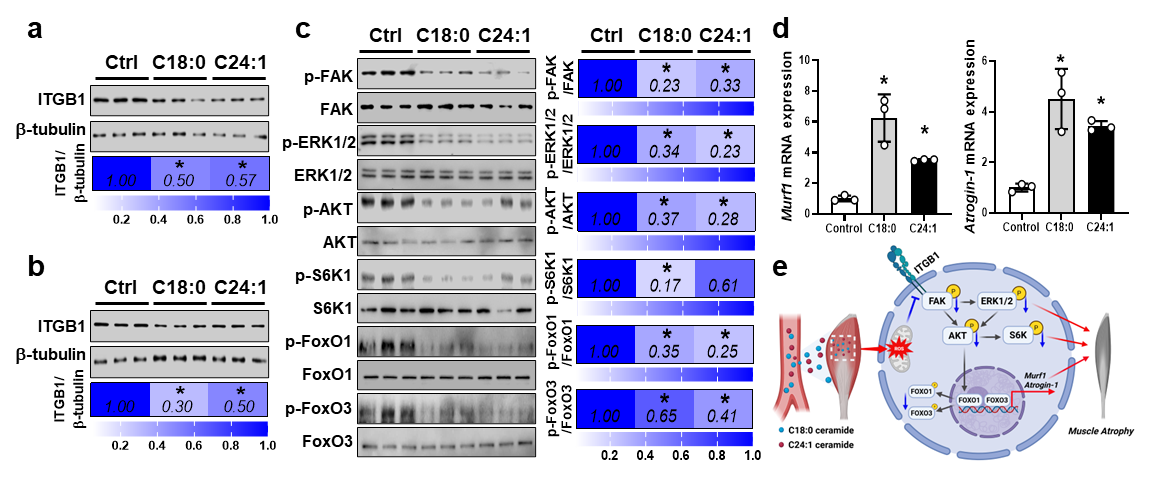
**

**Supplementary Figure 4.** Systemic ceramide exposure suppresses ITGB1 signaling and promotes muscle atrophy programs in vivo. (a, b) Western blot analyses were performed to assess ITGB1 protein expression in (a) tibialis anterior and (b) soleus muscles of mice treated with C18:0 or C24:1 ceramide (n = 3). (c) Western blot analyses were conducted to evaluate ITGB1-associated signaling pathways in soleus muscle tissues (n = 3). (d) mRNA expression levels of MuRF1 and Atrogin-1 in skeletal muscle tissues were assessed by quantitative reverse transcription polymerase chain reaction (n = 3). (e) Proposed model illustrating the mechanism. Created with BioRender. Wei, S. (2026) https://BioRender.com/k5k2e7c. An asterisk (*) indicates a statistically significant difference vs. untreated control (a–d).


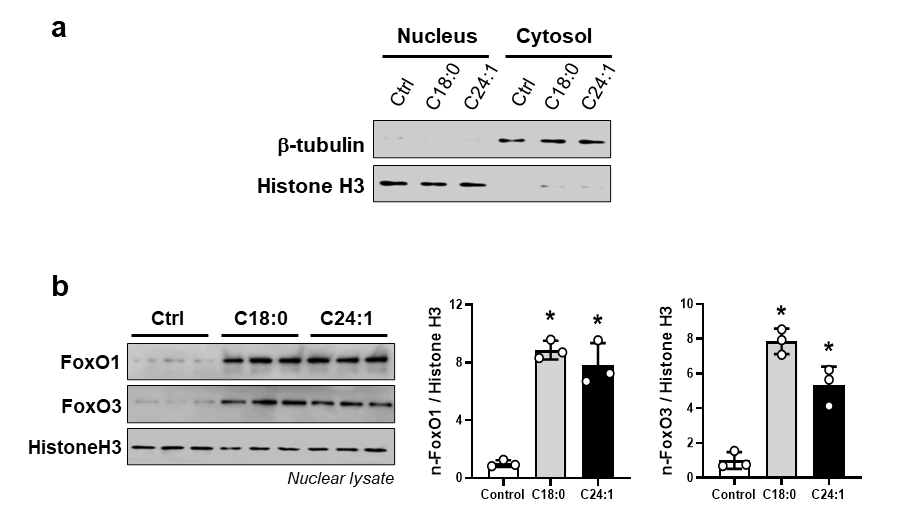


**Supplementary Figure 5**. C18:0 and C24:1 ceramides promote the nuclear accumulation of FoxO transcription factors in skeletal muscle. (a) Nuclear proteins were isolated from the skeletal muscle tissues of control and ceramide-treated mice. Western blot analyses were performed to assess β-tubulin and Histone H3 levels in the nuclear and cytosolic fractions. (b) Western blot analyses were performed to assess FoxO1 and FoxO3 levels in the nuclear fractions. Histone H3 served as a nuclear loading control (n = 3). * *P* < 0.05 vs. untreated control.
